# Effect of selective removal of badgers (*Meles meles*) on ranging behaviour during a ‘Test and Vaccinate or Remove’ intervention in Northern Ireland

**DOI:** 10.1017/S0950268821001096

**Published:** 2021-05-07

**Authors:** M. J. H. O'Hagan, A. W. Gordon, C. M. McCormick, S. F. Collins, N. A. Trimble, C. F. McGeown, G. E. McHugh, K. R. McBride, F. D. Menzies

**Affiliations:** 1 Department of Agriculture, Environment and Rural Affairs, Veterinary Epidemiology Unit, Upper Newtownards Road, Belfast, BT4 3SB, Northern Ireland, UK; 2 Agri-Food and Biosciences Institute, Statistical Services Branch, Newforge Lane, Belfast, BT9 5PX, Northern Ireland, UK; 3 Agri-Food and Biosciences Institute, Veterinary Sciences Division, Stoney Road, Belfast, BT4 3SD, Northern Ireland, UK; 4 Department of Agriculture, Environment and Rural Affairs, TVR Field Implementation Unit, Glenree House, Springhill Road, Newry, BT35 6EF, Northern Ireland, UK; 5 Department of Agriculture, Environment and Rural Affairs, Upper Newtownards Road, Belfast, BT4 3SB, Northern Ireland, UK

**Keywords:** Badgers, Bovine tuberculosis, homeranges, selective culling, test and vaccinate or remove

## Abstract

The role of the Eurasian badger (*Meles meles*) as a wildlife host has complicated the management of bovine tuberculosis (bTB) in cattle. Badger ranging behaviour has previously been found to be altered by culling of badgers and has been suggested to increase the transmission of bTB either among badgers or between badgers and cattle. In 2014, a five-year bTB intervention research project in a 100 km^2^ area in Northern Ireland was initiated involving selective removal of dual path platform (DPP) VetTB (immunoassay) test positive badgers and vaccination followed by release of DPP test negative badgers (‘Test and Vaccinate or Remove’). Home range sizes, based on position data obtained from global positioning system collared badgers, were compared between the first year of the project, where no DPP test positive badgers were removed, and follow-up years 2–4 when DPP test positive badgers were removed. A total of 105 individual badgers were followed over 21 200 collar tracking nights. Using multivariable analyses, neither annual nor monthly home ranges differed significantly in size between years, suggesting they were not significantly altered by the bTB intervention that was applied in the study area.

## Introduction

Bovine tuberculosis (bTB), caused by *Mycobacterium bovis*, has a serious economic impact on the cattle industry in the United Kingdom and Ireland, as well as elsewhere. It can affect most mammals including humans [1]. The role of wildlife as a host for *M. bovis* infection has been demonstrated globally with Eurasian badgers (*Meles meles*) being of importance in the British Isles [2]. Where the wildlife population is persistently infected, reduction of transmission can be difficult to achieve both within and between species [3]. The evidence base for badgers having a role in bTB epidemiology on the British Isles includes the known susceptibility of badgers for bTB [2], the plausibility of transmission routes (mainly via excretion from the respiratory tract, but also via excretions of the digestive and urinary tracts and exudates from skin lesions) [2], evidence based on molecular strain typing [4] and the outcomes of badger culling trials [5, 6].

As badgers are legally protected (Wildlife (NI) Order of 1985 [7] and Appendix III of the Berne Convention [8]), any interference with badgers and their setts is only allowed under licence. Surveillance for bTB in badgers has been taking place since 1998 in Northern Ireland in the form of a road traffic accident survey covering all areas of the province. Bovine TB infection was confirmed in badgers by bacteriological isolation [9]. This survey estimated the Northern Ireland badger bTB prevalence to be 15.3% (95% confidence interval 13.1–17.5) [9].

Badger populations with an artificially reduced density due to culling have been suggested to have increased badger movement leading to increased home range area sizes, which in turn has been hypothesised to increase the possibility of contact and bTB transmission between badgers and also between badgers and cattle [5, 10–12]. Previous research has suggested that even very low levels of culling may be sufficient to produce a measurable perturbation effect (i.e. a deviation of badger movement compared to its regular or normal path caused by an outside influence) [13]. In Britain, perturbation due to non-selective culling was followed by a reduction in cattle bTB incidence [11]. In the Republic of Ireland (RoI), findings suggested that a reduction in badger numbers contributed to the control of bTB in cattle without inducing perturbation [6, 14]. The reason perturbation was not recorded in RoI is suggested to be due to a variety of factors including culling efforts and geographical differences such as the configuration of cattle farms and badger social group size [15, 16].

An alternative bTB control option to culling is badger vaccination. Vaccination with Bacillus Calmette-Guerin (BCG) strains was shown to reduce the severity and progression of disease in naive badgers [17] and to indirectly reduce the incidence of disease in unvaccinated cubs [18]. A previous study found that the majority of farmers in a Northern Ireland context would allow badger vaccination and culling on their own land but the overall preference was for vaccination [19].

In 2014, a five-year bTB intervention research project was initiated in a 100 km^2^ area of County Down, Northern Ireland. The intervention involved selective removal of bTB test positive badgers and vaccination and release of bTB test negative badgers. This so called ‘Test and Vaccinate or Remove’ (TVR) approach involved a lower level of badger removal than non-selective culling and it was therefore considered more publicly acceptable [20]. Furthermore, a combination of selective removal of bTB infected badgers (rather than all captured badgers in non-selective culling interventions) in parallel with BCG vaccination to boost the immunity level of the remaining badger population should provide a synergy enabling accelerated control of bTB infection within the badger population [20].

A previous modelling study concluded that the likely benefit of selective culling will be dependent on whether or not perturbation occurs in the badger population [16]. The effect of selective culling of badgers in an area on badger movement is so far unknown. Thus the main aim of the study here described was to evaluate the effect on badgers’ home range sizes after the selective culling of bTB test positive badgers. This objective was achieved through fitting global positioning system (GPS) collars on a subset of badgers within the TVR project.

## Methods

### Study area

**Fig. 1.**
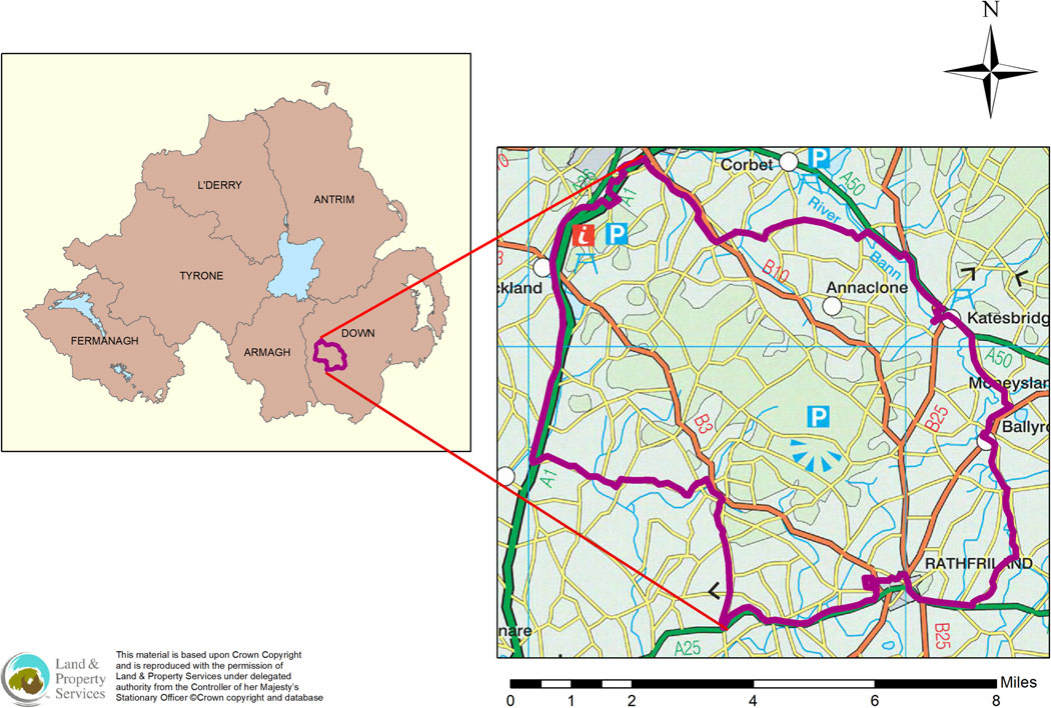
Location of the Test and Vaccinate or Remove zone in co. Down, Northern Ireland.

The area selection was based on high confirmed bTB cattle herd prevalence during a two-year period (2011 and 2012) (two-year confirmed TB prevalence 24%) as well as having high cattle (229 cattle herds; 168 cattle per km^2^) and active main badger sett densities (1.12 active main setts per km^2^). The area was selected based on recommendations after modelling conducted prior to TVR intervention [15]. The 100 km^2^ area was bounded by a busy dual-carriage way (main Belfast-Dublin road) and a river to its north-east (Fig 1), which formed relatively hard boundaries with the aim of reducing the immigration of badgers into the study area [15]. A preliminary badger sett survey was conducted prior to commencement of the study [21]. The study area consisted of grass land (67 km^2^) and some arable land (13 km^2^) with the remainder being woodland, residential areas and farm-yards.

### Study design and study population

The ranging behaviour, based on calculation of home range sizes, was measured using GPS collars fitted to a subset of badgers every year. Home ranges of wild animals like badgers are defined as the area that an animal commonly uses for normal activities [22]. For the purposes of this paper, badger home ranges were defined using various kernel density measures (see later).

Badger capture involved saturation trapping (deployment of more cages than the anticipated number of badgers present) with individual cage traps based on the methodology and training provided by the Animal and Plant Health Agency's (APHA) National Wildlife Management Centre (NWMC). Cage traps were deployed at setts (1–6 cage traps per sett depending on noted activity) and other sites with signs of badger activity (e.g. badger runs). Every trapping cycle took three weeks with area surveys and digging in of the cage traps taking place in the first week, pre-baiting with peanuts during the second week and trapping in the third week. In Northern Ireland, licences for intervening with badgers are only granted by the Northern Ireland Environmental Agency (NIEA) between 1 July and 30 November resulting in only a small proportion of captured badger sows still being in late stage lactation. Therefore for the current study field work commenced in June with trapping normally taking place from the start of July until mid/end October each year. The research project was licensed (licence number 2767) under the Animals (Scientific Procedures) Act 1986 (as amended) [23]. The Animal Welfare Ethical Review Body (AWERB) within the Department of Agriculture, Environment and Rural Affairs (DAERA) also oversaw the project. Licences were also obtained from Northern Ireland Environment Agency (NIEA) to allow the capture, sampling, GPS collaring and removal of badgers. Permissions from land owners for trapping on their land were obtained from 96% (*n* = 831) of land owners equating to 93% of the fields (*n* = 6006) in the zone.

Trapped badgers were anaesthetised by a veterinary surgeon using medetomidine hydrochloride (1 mg/ml), ketamine hydrochloride (100 mg/ml) and butorphanol tartrate (10 mg/ml) by intramuscular injection (at 0.25 ml/kg at a ratio of 2:1:2 by volume). Badgers were monitored throughout and ‘topped up’ when necessary. Animals were individually identified by implanting a microchip subcutaneously on their first capture. All captured badgers received detailed physical examination and blood samples were taken from the jugular vein to determine their bTB status using a cage side test (DualPath Platform (DPP) VetTB test, Chembio Diagnostics Systems Inc., Medford, NY 11763 USA). The DPP VetTB test is a single use, point of care, immune-chromatographic (lateral-flow) rapid test for the detection of antibodies to *Mycobacterium tuberculosis* and *M. bovis* in cervid serum. Whole blood was used to determine the badger's bTB status in the field [20, 24].

Tellus light GPS collar (Followit Wildlife, Lindesberg, Sweden; weight 240 g; on-board storage and global system for mobile communication (GSM) download) were fitted on a subset of trapped badgers. Once fitted, an attempt was made to remove the collar over the badger's head and tightened or removed if the badger was deemed unsuitable.

In order to facilitate a quick release into the field and prevent a prolonged recovery from anaesthesia or any other adverse effects (such as cardio-respiratory arrest), the effects of medetomidine were reversed with atipamezole (5 mg/ml) by intramuscular injection at a dose rate of 1/10th of the volume of triple anaesthetic cocktail (as described above).

GPS collars were fitted during the first four years of the TVR project as the fifth year focused on collar removal. The aim was to deploy 40 GPS collars (two badgers, preferably a male and a female, per social group) during each of the four years. Only badgers heavier than 8 kg and a cranial circumference of at least 1 cm more than the neck circumference, were deemed suitable to receive a GPS collar, similar to approaches taken in previous studies [25]. In cases where more than one badger was caught in a social group, the animal in best body condition, which had less potential to put on weight, was selected. GPS collars were not deployed where there was a wound, evidence of skin irritation around the neck or in the case where the neck was very muddy or wet. Furthermore, GPS collars were not fitted on badgers that were caught less than 1 km from the study area boundary. The GPS collars were programmed to record eight locations per night (hourly intervals from 21.00 to 04.00 h); similar to the settings used in other studies [25]. Location data were collected until the GPS collar stopped transmitting.

If badgers were recaptured in the same year, they were identified, recorded and released. Captured badgers that were fitted with GPS collars in previous years were fitted with a collar again provided the above criteria were met.

In the first year of the project (2014) all captured badgers were tested, vaccinated and released (captured badgers were released prior to results of the DPP test being known so test positive badgers were never knowingly released). In years 2–4, DPP test negative badgers were vaccinated and released while DDP test positive badgers were euthanised using pentobarbitone (140 mg/kg by intravenous injection). The vaccine used was Badger BCG (BCG Danish strain with 2–8 × 10^6^ colony forming units per dose; intramuscular administration) during years 1–3 and BCG Sofia (Intervax Ltd, Canada; BCG Sofia strain with 1.5–6.0 × 10^6^ colony forming units per dose; intramuscular administration) during the years thereafter [20].

Additional efforts were made to retrieve GPS collars that stopped transmitting by recapturing collared badgers with additional assistance provided by using motion-activated cameras. Collars were considered accounted for when badgers had their collars removed upon recapture, when collared badgers were found dead (e.g., as a result of a road traffic accident) and by identifying recaptured badgers that had lost their collar.

### Data analyses

#### Badger demography

A badger population estimate within the study area was obtained based on results from previous research [20, 26] and the Lincoln-Peterson Method using trapping data [27]. Distances between active main setts were calculated using the ‘Nearest Neighbour Tool’ in ArcGIS 10.3.1 (ESRI systems, USA) in order to provide background information into the badger density in the area. The number of trapped badgers removed each year was also recorded enabling the percentage of the estimated population removed as part of TVR project to be estimated annually.

#### Calculation of home ranges

Position data transmitted by GPS collars were used to determine home ranges of the collared badgers. These data were downloaded from the Followit website (https://www.followit.se/) and stored and manipulated in Microsoft Excel (version 2013; Microsoft Corporation, Redmond, WA, USA). Data were checked and cleaned. Data points that were based on <4 satellites to obtain the position were removed to ensure accuracy of the location points. Thereafter, data were transferred into ArcGIS (version 10.3.1; ESRI systems, USA) and R version 4.0.1 [28] where further manipulations and analyses were conducted.

Each animal's home range was defined using annual 95% and 50% fixed kernels (FK) [29, 30] constructed using their recorded GPS locations as were monthly 95% FK home ranges for every month they were collared. Kernel density calculates a magnitude per unit area from a point feature using a kernel function to fit a smoothly tapered surface to each point or polyline. A FK refers to a kernel where the bandwidth remains the same for all calculations. After applying kernel smoothing to the data points separately for every individual badger each year and for every individual badger for each month, probability contours were applied to created polygons reflecting the smallest area enclosing 95% or 50% of the probability distribution for the annual location points and polygons reflecting the smallest area enclosing 95% of the probability distribution for the monthly location points.

The home ranges were calculated using ArcGIS and results were compared to a subset of home ranges calculated using R (adehabitatR [31]) using the same location data and smoothing parameter *H* = 55. As the home range size estimates were not normally distributed (based on the Shapiro–Wilk normality test), annual and monthly median values were used for reporting descriptive overviews. For analyses, annual and monthly home ranges for each badger were taken forward.

#### Statistical analyses

Three analyses were performed. For the first two analyses, both 95% FK and 50% FK were constructed for every collared badger for each year they were captured based on all location points collected. In the first analysis, the outcome modelled was the size of the annual home ranges based on 95% FK whereas in the second analyses the outcome modelled was the size of the annual home ranges based on 50% FK. In the third analysis, the outcome modelled was the size of the monthly home ranges based on 95% FK.

A range of potential explanatory variables were examined in the analyses. The variable ‘*TVR year*’ (the year that a GPS collar was fitted on the badger) was evaluated in order to measure a potential correlation between year and home range size to examine the potential effect of intervention on home range size. The ‘*Number of locations points recorded per badger*’ was also evaluated as an explanatory variable along with ‘*Sex*’ of the badger, ‘*Cull intensity*’ (low/medium/high; taking into account a 1 km and 3 km radius around the GPS data points of culled badgers during the year based on constructed kernel densities) and ‘*Main sett density*’ (low/medium/high based on constructed kernel densities around location points of main setts based on a search radius of 1 km). This method of using kernel densities estimates the intensity of events across a surface by calculating the overall number of cases situated within a given search radius from a target point. This has been applied in previous studies, as an alternative to zonal approaches based on data aggregation and calculation rates, with the aim of preventing the reduction in the number of observation points and the probability of ecological bias [32]. The allocation of the culling and main sett density category were based on where the 50% FK area of the badger was situated in. ‘*Season*’ was added as an additional potential explanatory variable to the third analysis based on monthly home ranges. ‘*Season*’ was defined as follows: Winter (January–March), Spring (April–June), Summer (July–September), Autumn (October–December). If a monthly home range was calculated for all three months within a season then it had three measurements to reflect this.

The analyses were conducted using GenStat (version 18; VSNI, Hemel Hempstead, UK). For each response variable, a list of candidate variables was supplied as possible explanatory variables. In each case a stepwise regression (forward selection with backward elimination) analysis approach was implemented in order to select the ‘best’ model for the response variable in question. Each of the models generated at this stage was then refitted using a linear mixed model methodology with individual badger (‘*Badger ID*’) and *‘TVR year*’ fitted as a random effect and the rest of the previous named variables as fixed effects [33]. Doing this ensured that repeated observations on the same badger over years/months are nested within *‘Badger ID*’ and consequently have a uniform correlation structure applied to them. This step ignored the random structure of the study and so the proper random structure was introduced at this point and a backwards elimination procedure then applied to pick the final ‘best’ model so that only explanatory variables that were significant (*P* < 0.05) were left in the final model in each case [34]. This approach of linear mixed model methodology assumes normality of residuals. This as well as other requirements e.g. homoscedasticity of residuals etc. was assessed by visual inspection of the appropriate residual plots.

The differences in the size of the home ranges in the first year between males and females based on annual 95% FK were evaluated using the independent *t*-test. Correlation analyses were conducted between ‘Number of location points recorded per badger’ and home range sizes using Pearson correlation coefficients. Outcomes of home range size calculations using ArcGIS and R were compared using the two sample *t* test.

## Results

The population density estimate for the study area, based on previous research [20, 26] and the Lincoln Peterson method [27], was 5.6 badgers/km^2^ with the total population estimate being 560 badgers. The number of trapped badgers removed ranged from 11 to 56 per year during years 2–4 (2015–2017) which represented between 2.0–10.0% of the estimated population (*n* = 560) (Table 1). The mean distance between adjacent active main setts was calculated to be 820 m and followed a dispersed (non-clustered) pattern (*z*-score = 5.042; *P* value < 0.001). The vast majority (89%) of GPS collars were recovered/accounted for after they stopped transmitting (117 (75%) were recovered at next capture, 11 (7%) were recovered from road traffic accident badgers and 10 (6%) badgers were recaptured but had lost their collars prior to recapture).

**Table 1.** Number and percentage of badgers trapped and removed during the Test and Vaccinate or Remove project

Year	Badgers trapped	Badgers removed	% of trapped badgers removed	% of estimated population removed^a^
2014	280	0	0	0
2015	341	56	16.4	10.0
2016	271	11	4.1	2.0
2017	287	22	7.7	3.9
2018	341	19	5.6	3.4

a Based on an estimated population of 560 badgers [20].

**Fig. 2.**
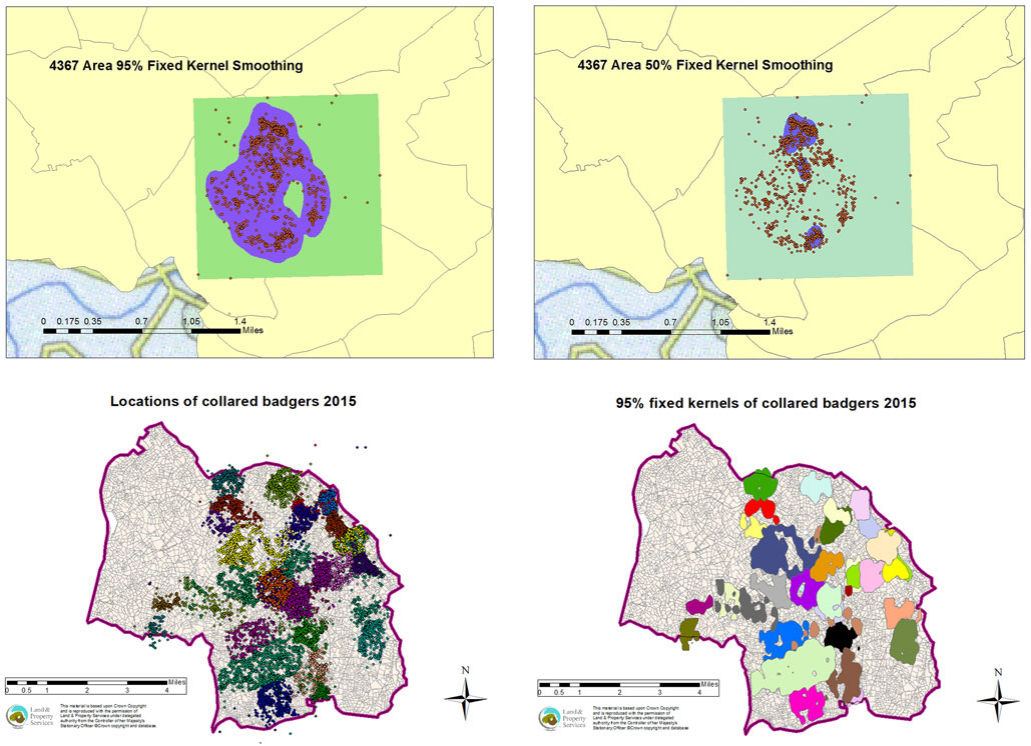
Explanatory map with an example of the 95% and 50% FK creation for badger 4367, an example of all location points in one year (2015) and an example of all 95% annual fixed kernels in one year (2015).

**Fig. 3.**
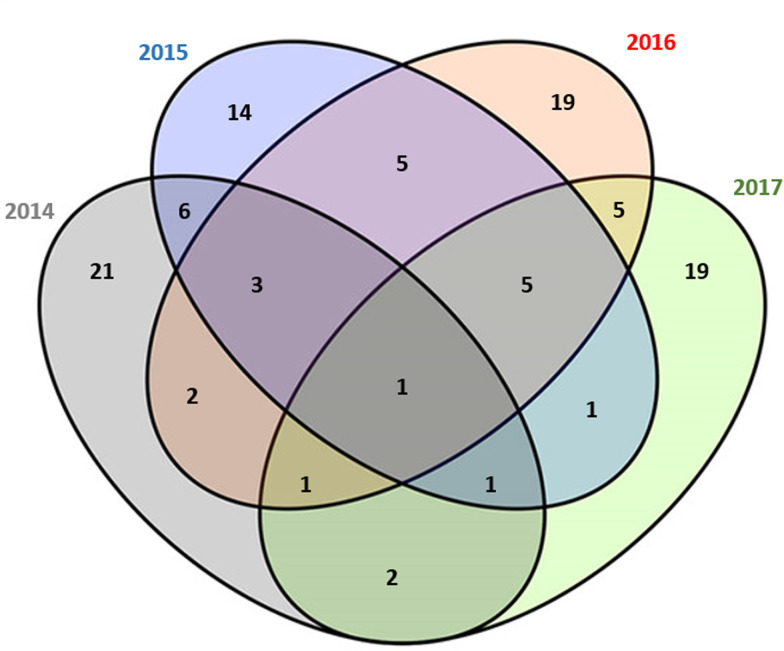
Venn diagram in relation to the number of times badgers were collared in the Test and Vaccinate or Remove study (2014–2017).

The descriptive results of the badgers collared in years 1–4 (2014–2017) are displayed in Table 2 with explanatory maps displayed in Figure 2. The number of badgers that were fitted with a GPS collar and transmitted their location points successfully varied from 35 to 41 per year. Over the TVR study period, fewer female badgers were collared than male badgers (overall ratio 1:1.44). The majority of location points were recorded during the summer (43.0%) and autumn (45.0%), whereas the remainder were recorded during the winter (8.6%) and spring (3.4%). Badger ranging behaviour was analysed based on home range estimates from 105 individual badgers over 21 200 collar tracking nights. One badger was collared all 4 years, 10 badgers were collared 3 out of 4 years, 21 badgers were collared 2 out of 4 years and 73 badgers were only collared once during the study period (Fig. 3). Minor neck injuries were recorded in two adult badgers due to the GPS collars applied [20].

**Table 2.** Descriptive results of badgers collared in the Test and Vaccinate or Remove zone (2014–2017)

Year collar was fitted	2014	2015	2016	2017
Total number of badgers collared	37	36	41	35
Number of cubs collared	2	0	1	2
Number of female badgers collared	17	17	13	14
Number of male badgers collared	20	19	28	21
*Ratio female:males*	*1:1.18*	*1:1.12*	*1:2.15*	*1:1.50*
Number of days of recording per badger				
Median	176	132	152	104
Range	20–337	11–303	26–302	18−151
Q1–Q3^a^	99–241	81–217	118–182	62−131
Number of locations recorded per badger				
Median	909	753	887	724
Range	108–2132	76–1947	101–1921	130–1050
Q1–Q3^a^	667–1363	548–973	709–1094	399–898

a 25th percentile–75th percentile.

Home ranges derived using ArcGIS and R produced similar results (paired samples *t*-test; *t* = 1.2829, *P* = 0.2259), hence only ArcGIS outputs were used to obtain the subsequent data presented in these analyses. Descriptive summaries of the home range sizes for each year based on the previously defined FKs are presented in Table 3. The sample size was 536 monthly home ranges over a four-year period (2014–2017). Based on data collected in year 1 (2014), male badgers ranged over a larger area than female badgers although this was not statistically significant (based on annual 95% FK; *t* = −1.4787, df = 36, *P* = 0.148; median home range size for males and female being 1.67 and 1.35 km^2^, respectively). There was a weak positive correlation between the number of locations and the size of the home ranges (*r* = 0.381 for annual 95% FK; *r* = 0.278 for annual 50% FK).

**Table 3.** Home ranges based on annual 95% fixed kernels (FK), annual 50% FK and monthly 95% FK of badgers collared during the Test and Vaccinate or Remove project (2014–2017)

Year collar was applied	2014	2015	2016	2017
Annual 95% FK^a^ (km^2^)				
Median	*1.41*	*1.15*	*1.36*	*1.22*
Range	0.51–3.94	0.11–4.82	0.15–3.81	0.46–2.30
Q1–Q3^b^	1.02–2.06	0.82–1.50	0.84–1.61	0.79–1.55
Annual 50% FK^c^ (km^2^)				
Median	*0.15*	*0.10*	*0.14*	*0.13*
Range	0.05–0.51	0.04–0.34	0.04–0.40	0.02–0.32
Q1–Q3^b^	0.11–0.22	0.07–0.16	0.09–0.19	0.08–0.17
Monthly 95% FK^a^ (km^2^)				
Median	*1.08*	*0.89*	*0.98*	*0.92*
Range	0.26–3.07	0.11–3.76	0.08–2.79	0.10–2.51
Q1–Q3^b^	0.80–1.33	0.58–1.25	0.68–1.30	0.65–1.19

a 95% Fixed kernel.

b 50% Fixed kernel.

c 25th percentile–75th percentile.

The year the GPS collar was fitted (‘*TVR Year*’; a proxy for the effect of TVR intervention) was not a significant explanatory variable in the multivariable analyses. However, the results showed that annual home ranges were significantly associated with the ‘*Number of locations recorded per badger*’ while ‘*Sex*’ was only a significant factor in the annual home ranges based on 95% FK. For the monthly 95% FK home ranges ‘*Season*’, ‘*Sex*’ and ‘*Main sett density*’ were all significant factors (Table 4). ‘*Culling intensity’* was offered as a candidate variable but not selected in the final model.

**Table 4.** Multivariable analyses results based on the response variable home range size

	Variable	Estimate	S.E.^a^	*P*
Annual 95% FK (km^2^)	Constant	63.73	13.382	
	Number of locations recorded per badger	0.068	0.0116	<0.001
	Sex			
	Female	0.000	13.070	0.017
	Male	31.59		
Annual 50% FK (km^2^)	Constant	8.738	1.508	
	Number of locations recorded per badger	0.007	0.002	<0.001
Monthly 95% FK (km^2^)	Constant	110.8	11.29	
	Season^b^			
	Winter	0.00	6.799	<0.001
	Spring	9.79		
	Summer	−20.00		
	Autumn	−34.40		
	Sex			
	Female	0.00	8.169	0.004
	Male	24.05		
	Main sett density			
	Low	0.00	11.620	0.041
	Medium	6.954		
	High	−22.299		

a Standard error.

b Winter (January–March), Spring (April–June), Summer (July–September), Autumn (October–December).

## Discussion

Where wildlife acts as a potential host for infectious diseases, their home range sizes are important in relation to transmission dynamics [10]. In the case of badgers, which are widely accepted as a wildlife host for bTB infection in the British Isles [2], knowledge of home ranges is especially of importance. This is because previous research conducted in England has suggested that badger culling could lead to social perturbation with a subsequent perturbation effect of increased prevalence levels of bTB in cattle in surrounding areas [10]. This social disruption (perturbation effect) may influence contact rates between badgers, potentially increasing disease transmission in the population and/or the likelihood that infected animals become infectious through stress-induced immuno-suppression [35].

The intervention trial described in the current study is a unique approach that has never been conducted elsewhere. Therefore, the effects of this approach on badger ranging behaviour were unknown. Badger ranging behaviour has previously been suggested as one of the factors, alongside trapping efficiency, vaccine efficacy and test performance, which could impact on the disease control benefits of the TVR approach [16].

The most straightforward method to estimate a home range is construction of the smallest possible convex polygon (100% minimum convex polygon (100% MCP)), which calculates the area enclosing all recorded GPS data points for every collared badger each year. Although easily constructed, 100% MCP have important short comings as the density of the data points is not taken into account with this method [22] and therefore can lead to an overestimation of the home range size [36] and therefore were not considered for this study. Home ranges based on 95% fixed kernels are probably the most accurate estimator of the area that animal uses during its normal activities [29] as it excludes outlier locations. Home ranges sizes based on 50% FK are condensed even more and often only focus on the core area an animal uses [37].

We analysed the badger ranging behaviour based on home range estimates from 105 individual badgers over 21 200 collar tracking nights. As the badger density in our study area is estimated to be in the band 3.0–9.99 individuals/km^2^, it is considered to be high by Gaughran *et al*. [25]. Population density is known to be highly, negatively correlated to home range size with the mean home range size for high badger population density areas estimated to be 0.77 km^2^ (s.e. ± 0.15) [25]. Referring to the home ranges calculated in the base line year (year 1; 2014) of the current study, our home ranges were larger (median 1.41 km^2^). However, it has to be kept in mind that the current study also shows that the number of locations measured was found to be positively associated with the size of the home range and that home range size depends on the method used [38]. Moreover, the home range estimates in the current study are very similar to recent research conducted in Ireland [25] where a mean home range size of 1.4 km^2^ was quoted, but again as different methods are used this figure is not directly comparable [39].

In the multivariable analyses, the year of study (‘*TVR Year*’ was used as a proxy for the effect of the intervention as intervention took place in the last 3 years (2015–2017) of the study. In all of the analyses conducted, ‘*TVR Year*’ was not an explanatory variable that had a significant influence on the outcome variable (annual and monthly home range sizes) suggesting that the home ranges did not significantly differ in size between years or therefore were not significantly altered by the bTB intervention/badger removal that was applied in the study area. This finding did not only apply to the home range sizes based on annual location points, but also when monthly home range sizes were taken into account.

‘*Culling intensity*’ was offered as a candidate variable but not selected in the final model. It has to be kept in mind that in the current study only a small proportion of badgers (2.0–10.0%) were removed annually. Currently it is unknown whether there is a threshold in terms of social group members removed [16], however, previous research, based on data modelling, indicated that removal of even one badger may induce perturbation [13]. With a selective culling approach, based on TVR intervention, the proportion of badgers being removed have been substantially reduced compared to a non-selective culling approach (70% removal based on 4–7 culling years [39]), which based on the current analyses conducted did not induce increased ranging behaviour. Indeed, there are more badgers removed from the area by road traffic collision fatalities than through the intervention in the current study [9, 40]. It is known for example that during 2016, 29 road traffic fatalities were reported in the TVR area while only 11 badgers were removed through the intervention in the same year. However, it has to be kept in mind that the effect of TVR operates in addition to the effects of road traffic fatalities and that it is not known whether road traffic fatalities in sufficient numbers would cause behaviour change among the survivors.

As the sensitivity of the DPP for use in badgers during the TVR study has recently been quoted as 42% (95% 0.24–0.66) [24] (and the specificity as 89% (95% CI 86–92%), [24]), there is a possibility that the number of DPP positive badgers, and therefore culled badgers, is low due to non-perfect sensitivity. However, as the performance of the DPP VetTB test is comparable to established laboratory tests for *M. bovis*, it is therefore an appropriate method to use under field circumstances [24]. Further consideration needs to be given to whether the DPP test can differentiate between natural exposure to *M. bovis* and previous vaccination. This is potentially one of the limitations of a TVR approach as the distinction between immunity due to natural infection and vaccination is of importance in the evaluation of the TVR concept.

The number of locations per badger was a significant explanatory variable in relation to the annual home range sizes, with an increase in the number of locations relating to an increase in the home range size. This was only a weak positive correlation that plateaued when high numbers of location points were reached. These findings are in line with previous research [41] that stated that in the case of territorial animals, such as badgers, a saturated sample size can be reached.

The sex of badgers was a significant explanatory variable in the multivariable analyses based on the outcome variable annual and monthly 95% FK home range size with male badgers tending to have larger home range sizes. This outcome concurred with previous research findings in Ireland [25]. A high main sett density was significantly associated with a smaller monthly home range size. This finding is also in line with previous research [42] and is directly linked to habitat characteristics with land most suitable for badgers (such as lowland park land with mixed woodland) reflecting a higher badger density and smaller home range sizes (and vice versa). The season was significantly associated with monthly home range size, with ranging behaviour being smallest in the autumn. This has previously been linked to earthworms being more abundant in autumn compared to any other season resulting in badgers being able to collect food from a smaller area. A lack of reproductive activity and a building of fat reserves have also been suggested as a further explanation [43].

There are some limitations to this study. Firstly, badgers were selected for suitability for collaring based on criteria relating to neck size and weight. This creates a bias in selecting larger badgers in the population (i.e. bias towards adult male badgers, which tend to have larger home ranges) for collaring. Furthermore it is worthwhile to note that, along with monthly variations in the number of location points collected per badger, in year 4 (2017) of the project a drop in recordings was noticed due to apparent premature failure of refurbished collars. The adjustment for number of locations in the analyses should however have taken account of both biases.

It is recommended that the described TVR approach would be repeated using replication and control areas (i.e. no intervention) as the current approach without these limits the interpretation of the study findings and its extrapolation to other areas. This recommended future research would help to validate the current findings [20].

This paper is a description of the first research into the potential changes in badger home range sizes after intervention based on a test and vaccinate or remove approach. This approach resulted in 2.0–10.0% of badgers being removed each year. Using a sample size of 536 monthly home ranges over four years, no significant alteration of ranging behaviour was found due to the TVR approach. This finding is important for informing on the development of future bTB intervention strategies. The non-significant impact of a TVR approach on badger ranging behaviour, alongside the previously described logistics of a large-scale TVR intervention [20] and the feasibility of the DPP test [24], gives a holistic overview of the suitability of TVR as a wildlife-related bTB intervention option. By providing an alternative to culling all captured badgers, and inevitably healthy ones, TVR is a wildlife intervention option that should be considered for future research using replication and control areas in order to verify the current findings.

## Data Availability

All materials needed to replicate the findings of the article are available as Supplementary Materials.

## Supplementary material

For supplementary material accompanying this paper visit http://dx.doi.org/10.1192/S0950268821001096. click here to view supplementary material
